# Estrogen Deficiency Induces the Differentiation of IL-17 Secreting Th17 Cells: A New Candidate in the Pathogenesis of Osteoporosis

**DOI:** 10.1371/journal.pone.0044552

**Published:** 2012-09-10

**Authors:** Abdul M. Tyagi, Kamini Srivastava, Mohd Nizam Mansoori, Ritu Trivedi, Naibedya Chattopadhyay, Divya Singh

**Affiliations:** Division of Endocrinology, Central Drug Research Institute (Council of Scientific and Industrial Research), Chattar Manzil, Lucknow, India; University Hospital Jena, Germany

## Abstract

Th17 cells produce IL-17, and the latter promotes bone loss in collagen-induced arthritis in mice. Blocking IL-17 action in mouse model of rheumatoid arthritis reduces disease symptoms. These observations suggest that Th17 cells may be involved in the pathogenesis of bone loss. However, the role of Th17 cell in estrogen (E2) deficiency-induced bone loss is still not very clear. We investigated the effect of E2 on Th17 differentiation *in vivo* and IL-17 mediated regulation of osteoclast and osteoblast differentiation. Additionally, effect of IL-17 functional block under E2 deficiency-induced bone loss was studied. In murine bone marrow cells, E2 suppressed IL-17 mediated osteoclast differentiation. IL-17 inhibited formation of mineralized nodules in osteoblasts and this effect was suppressed by E2. E2 treatment to mouse calvarial osteoblasts inhibited the IL-17-induced production of osteoclastogenic cytokines and NF-kB translocation. In ovariectomized mice, there was increase in the number of Th17 cells, transcription factors promoting Th17 cell differentiation and circulating IL-17 levels. These effects were reversed by E2 supplementation. Treatment of neutralizing IL-17 monoclonal antibody to Ovx mice mitigated the E2 deficiency-induced trabecular bone loss and reversed the decreased osteoprotegerin-to-receptor activator of nuclear factor kappa B ligand (RANKL) transcript levels in long bones, increased osteoclast differentiation from the bone marrow precursor cells and decreased osteoblast differentiation from the bone marrow stromal cells. Our findings indicate that E2 deficiency leads to increased differentiation of Th17 cells with attendant up regulation of STAT3, ROR-γt and ROR-α and downregulation of Foxp3 which antagonizes Th17 cell differentiation. Increased IL-17 production in turn induces bone loss by increasing pro-osteoclastogenic cytokines including TNF-α, IL-6 and RANKL from osteoblasts and functional block of IL-17 prevents bone loss. IL-17 thus plays a critical causal role in Ovx-induced bone loss and may be considered a potential therapeutic target in pathogenesis of post menopausal osteoporosis.

## Introduction

A relationship between the immune system and bone has long been speculated as bone loss is an invariable pathology of autoimmune and inflammatory conditions [1], [2], [3]. Osteoclasts are the bone resorbing cells, whose enhanced action due to inflammatory conditions is the pathology of bone loss [4], [5], [6]. T cells are key inducers of bone wasting under estrogen deficiency because ovariectomy (Ovx) increases the production of tumor necrosis factor-alpha (TNF-α) by T cells to a level sufficient to augment osteoclastogenesis via the increase in receptor activator of nuclear kappa B ligand (RANKL) [7], [8], [9], [10], [11], [12], [13], [14].

T cells have recently been shown to produce cytokines that could not be classified according to the Th1–Th2 system [15], [16], [17]. Interleukin-17 (IL-17) was among these cytokines, and the T cells that more selectively produce IL-17, but not interferon-γ or interleukin-4, were named Th17 cells [18]. Because these T cells constitute a distinct lineage, Th17 cells are now the third type of effector helper T cells in addition to Th1 and Th2 [19].

IL-17 has been shown to be an important mediator of inflammatory arthritis and other diseases affecting the bone [15]. For example, IL-17 was found to be highly elevated in synovial fluid from RA and osteoarthritis patients [20]. In addition, IL-17 has been implicated in the pathogenesis of RA in animal models [20]. IL-17 deficient mice are resistant to collagen-induced arthritis (CIA) and blocking IL-17 in a mouse CIA model reduced disease symptoms whereas excess IL-17 exacerbated disease conditions [16], [17], [21], [22]. Although IL-17 is implicated in bone erosion in RA, this cytokine plays a dominantly protective role in bone loss following periodontal infection [23]. Furthermore, in TallyHo/JngJ (TH) mice, a polygenic model of type II diabetes, IL-17 appears to mediate the bone loss [24]. These findings have important implications for the use of pharmacologic blockers of IL-17, as well as defining the *in vivo* biology of this cytokine.

**Figure 1:**
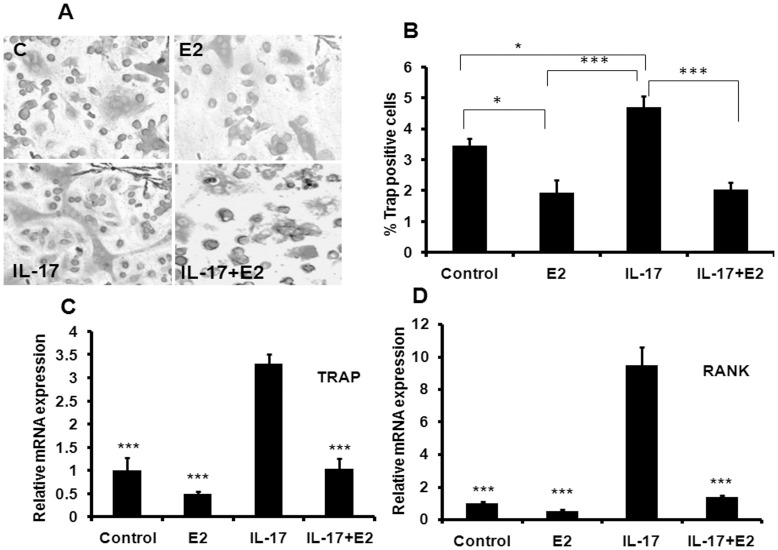
Estrogen prevents IL-17 induced differentiation of osteoclasts from BMCs. (A) Representative photomicrograph (20x magnification) show that IL-17 at 100 ng/ml concentration induced osteoclastogenesis from BMCs in presence of M-CSF (10 ng/ml) and RANKL (50 ng/ml) in five days culture. E2 treatment at 10^−9^M concentration inhibits IL-17 induced formation of multinucleated cells. (B) Quantitative representation of TRAP^+^ cells at various treatment conditions. (C) mRNA level of TRAP gene, which is a marker of functional osteoclast was determined by qPCR from the total RNA isolated from cultured cells. (D) RANK expression help in the differentiation of osteoclast precursor cells in to mature osteoclast. mRNA level of RANK gene was determined by qPCR from the total RNA isolated from cultured cells. Data represent three independent experiments and expressed as mean ± SEM with 95% confidence interval. Statistical analysis was performed by ANOVA method followed by the Newman–Keuls test of significance using Prism version 3.0 software.

Because of these compelling evidences in favour of bone loss caused by IL-17, monoclonal antibody against interleukin-17 has been developed for clinical application. Phase 2 trials of one such antibody against IL-17 (AIN457) for RA, Crohn’s disease, and psoriatic arthritis are under way [25]. However, the role of IL-17 under estrogen deficiency is not very clear. While the studies by Goswami et al, 2009 [26] have shown that Ovx IL-17 receptor knockout mice were more susceptible to bone loss than controls thus suggesting a bone protective role for IL-17 receptor signalling, contrasting observations by our group [27] and DeSelm et al [28] suggested that IL-17 mediates bone loss in estrogen deficient osteoporotic condition.

Thus, this study has investigated (i) the role of E2 in IL-17 regulated differentiation of murine osteoclasts and osteoblasts, (ii) proliferation of IL-17 secreting Th17 cells in Ovx induced bone loss condition, (iii) effect of E2 deficiency on factors regulating Th17 cell differentiation (iv) effect of functional block of IL-17 cytokine in Ovx-induced bone loss.

## Results

### Effect of E2 on IL-17 Induced Osteoclastogenesis

Effect of IL-17 on osteoclast differentiation in the presence of RANKL and MCSF was determined using murine bone marrow cells (BMC). Fig. 1 shows the representative image of TRAP positive cells and qPCR results of RANK and TRAP mRNA levels in BMC cultures treated with or without IL-17. Presence of IL-17 at 100 ng/ml in the culture stimulated the formation of multinucleated cells (P<0.05) (Fig. 1A and B) compared to control (without IL-17). However, IL-17 at 10 ng/ml concentration was found to be ineffective (data not shown). IL-17 induced osteoclastogenesis was inhibited by pre-treatment of BMCs with E2 (10^−9^ M) (P<0.001). Furthermore, IL-17 augmented the mRNA levels of RANK (P<0.001) and TRAP (P<0.001) compared to the control (Fig. 1C and D), while pre-treatment with E2 in IL-17 treated cultures reduced the expression of both genes.

**Figure 2:**
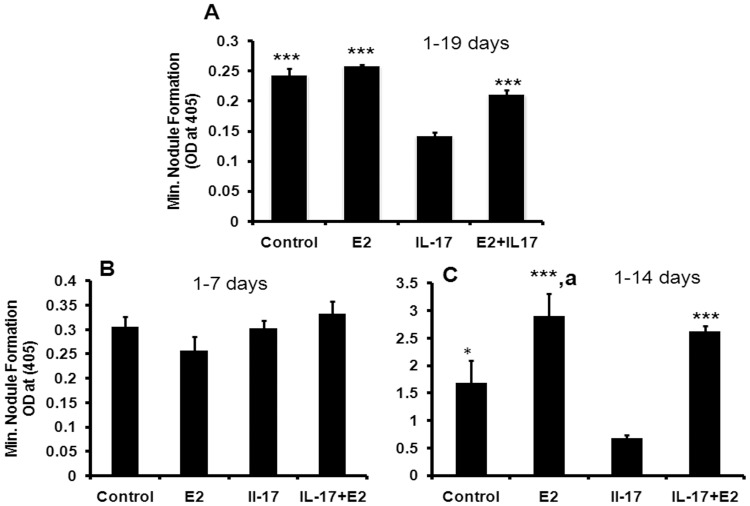
Estrogen prevents the IL-17 mediated reduction in mineral nodule formation. (A) Effect of E2 on IL-17 mediated inhibition of mineralization in 19 days cultured MCO. MCO was retrieved from 1–2 days old mice pups by sequential digestion with collagenase/dispase enzymes using standard method as given in material and methods. Quantitative measurement of mineralization nodules in all the groups were performed by using alizarin red staining. (B) Effect of E2 and IL-17 on MCO in 7 days mineralization. (C) Effect of E2 and IL-17 on MCO in 14 days mineralization. Data represent three independent experiments and expressed as mean ± SEM with 95% confidence interval. Statistical analysis was performed by ANOVA method followed by the Newman–Keuls test of significance using Prism version 3.0 software.

### Effects of E2 on IL-17 Mediated Inhibition of Osteoblast Differentiation

Continuous treatment of IL-17 for 19 days inhibited the mineralization of MCO (Fig. 2A) (P<0.001). We evaluated the effect of IL-17 on early (1–7 days) (Fig. 2B) and late stages (1–14 days) of formation of mineralized nodule by osteoblasts. IL-17, when added at the early stage had no effect but at the late stage, inhibited the formation of mineralized nodules (P<0.001) (Fig. 2C). E2 reversed the IL-17-induced inhibition of mineralization (P<0.001) (Fig. 2A–C).

### Effects of E2 on IL-17 Mediated Induction of Osteoclastogenic Cytokines and NF-κB Translocation

**Figure 3:**
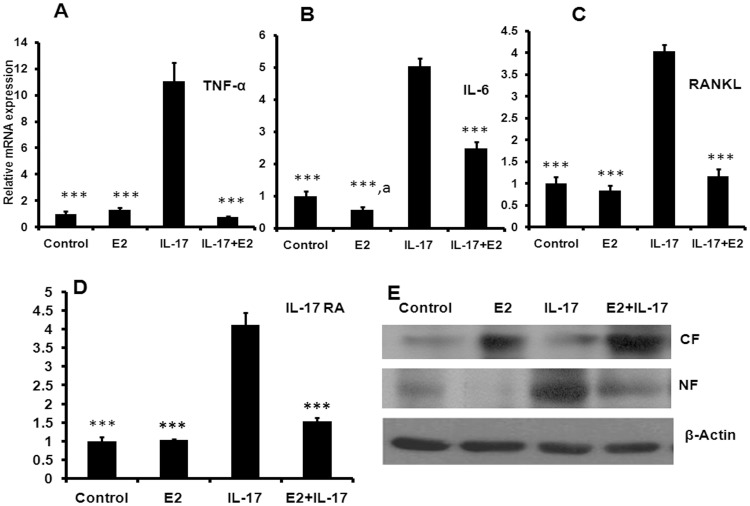
Estrogen prevents the IL-17 induced expression of osteoclastogenic cytokines in osteoblasts. (A) Effect of E2 on IL-17 induced osteoclastogenic cytokine TNF-α was evaluated. (B) Effect of E2 on IL-17 induced IL-6 mRNA level. (C) Effect of E2 on IL-17 induced RANKL mRNA level. (D) Effect of E2 on IL-17 induced IL-17RA mRNA level. (E) Effect of E2 on IL-17 induced NF-kB translocation. Data represent three independent experiments and expressed as mean ± SEM with 95% confidence interval. Statistical analysis was performed by ANOVA method followed by the Newman–Keuls test of significance using Prism version 3.0 software.

IL-17 induces the production of osteoclastogenic cytokines like TNF-α [25], [50]. Thus, we evaluated the effect of E2 on IL-17 induced osteoclastogenic cytokines production in MCO. IL-17 treatment to osteoblast cells led to robust increase in mRNA levels of TNF-α (P<0.001) (Fig. 3A), IL-6 (P<0.01) (Fig. 3B), RANKL (P<0.001) (Fig. 3C) and IL-17R (P<0.001) (Fig. 3D) while E2 was able to block the IL-17 induced expression of these osteoclastogenic factors.

IL-17 has been shown to induce expression of several cytokines known to contain nuclear factor kappa B (NF-*k*B) binding sites in their promoter [51]. Hence, it was studied whether E2 inhibits IL-17 mediated NF-κB signaling in osteoblasts. Using mouse MCO, we showed characteristic cytoplasmic and perinuclear localization of the NF-κB p65 subunit as observed in unstimulated osteoblasts (Fig. 3E). Treating osteoblasts with IL-17 resulted in predominantly intense nuclear labeling of p65 subunit (Fig. 3E). E2 inhibited IL-17stimulated nuclear translocation of p65 subunit.

### Effects of Ovx Induced Estrogen Deficiency on Th-17 Cells Proliferation and Circulating IL-17 Levels

**Figure 4:**
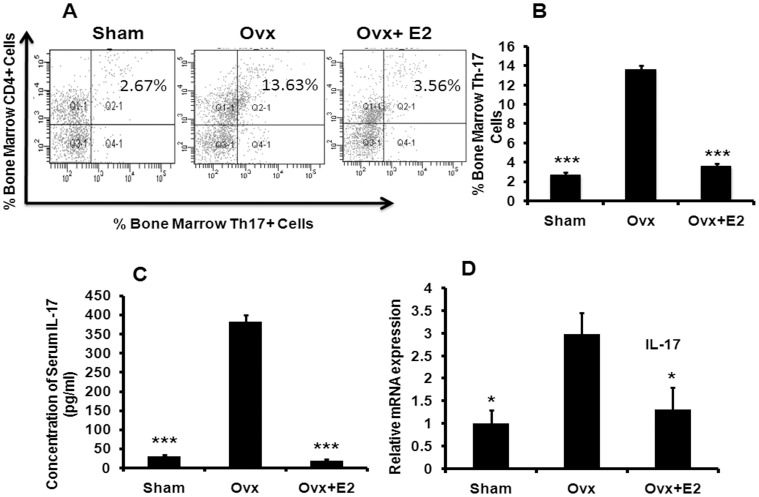
Ovx induces the production of IL-17 secreting Th17 cells, circulating IL-17 and transcript levels of IL-17. (A) Bone marrow cells were stimulated with 20 ng/ml PMA +250 ng/ml ionomycin in the presence of 2 µM monensin; then IL-17-producing cells were detected by FACS. Ovx induces the proliferation of Th17 cells compared to the sham control group (P<0.01). Oral administration of E2 at 0.01 mg/kg body wt reduces the number of Th-17 cells. (B) Bar diagram represents the number of Th-17 cells as percentage of total cells acquired in flow cytometer. Ten mice per group were taken for the study. (C) Serum level of circulating IL-17 was determined by specific ELISA as described in Materials and Methods. Each group represents results from a pool of eight mice. (D) Relative mRNA expression of IL-17 in isolated (detailed method of isolation was given in material and method section) CD4^+^ cells was measured in all the groups. CD4^+^ cells of five animals were pooled in each group for RNA isolation and to run real-time PCR assay. Data represent three independent experiments and expressed as mean ± SEM with 95% confidence interval. Statistical analysis was performed by ANOVA method followed by the Newman–Keuls test of significance using Prism version 3.0 software.

Because the major source of IL-17 is Th17 cells, we studied the effect of E2 deficiency on Th17 differentiation using Ovx mice. Ovx mice show significant loss in trabecular microarchitecture compared to sham group while E2 supplementation reverses this loss (Table S1). The Ovx group had higher population of CD4^+^IL-17^+^ cells (P<0.001) in the bone marrow compared to the sham (control) (Fig. 4A, B). E2 treatment to Ovx mice presented with decreased CD4^+^IL-17^+^ cells (P<0.001) (Fig. 4A, B). Circulating IL-17 levels were elevated in the Ovx group (P<0.001) compared to the control, however there was no difference in the IL-17 levels between the control and Ovx + E2 groups (Fig. 4C). Additionally, transcript levels of IL-17 in purified CD4^+^ cells from the bone marrow was determined. While IL-17 mRNA expression was elevated in Ovx animals (P<0.05), it was decreased and not different between sham and Ovx + E2 group (Fig. 4D).

### Effect of Ovx Induced Estrogen Deficiency on Factors Regulating Th17 Cell Differentiation

**Figure 5:**
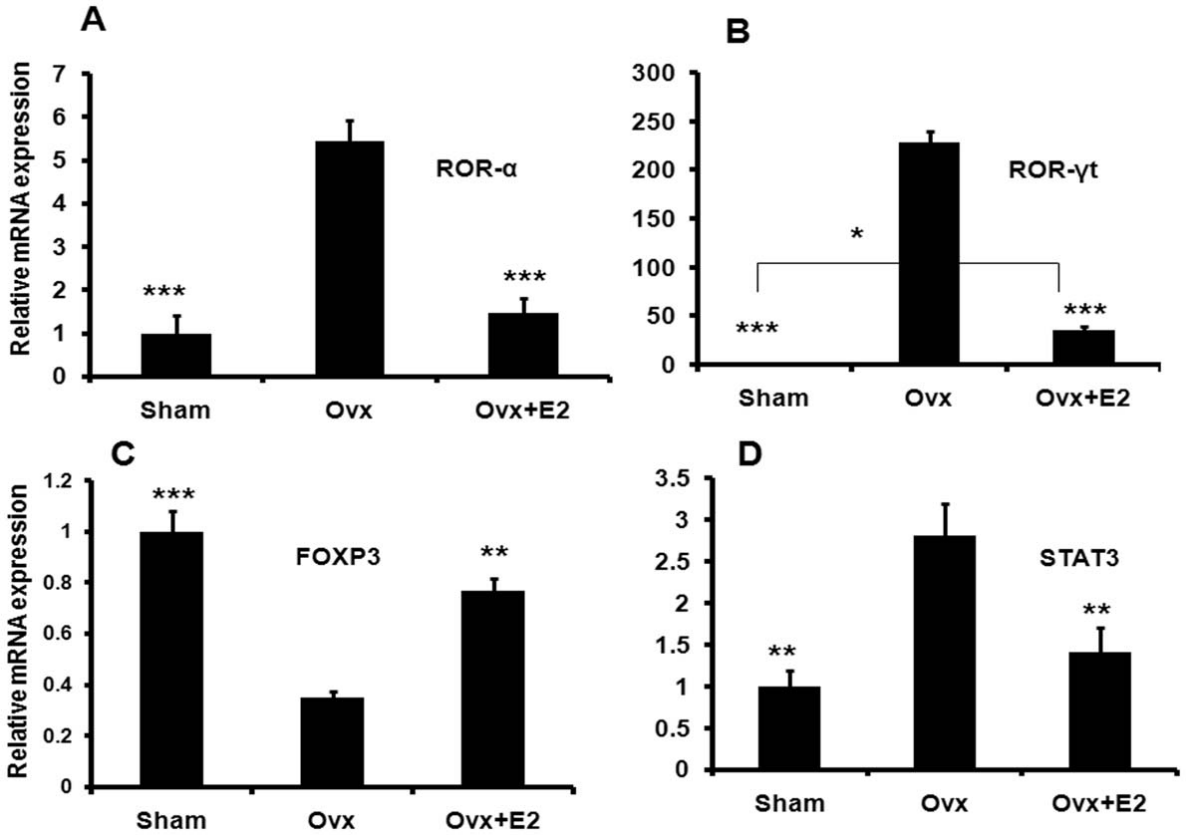
Ovx induces the transcript levels of factors regulating Th17 differentiation. Relative mRNA expression of ROR-α (A), ROR-γt (B), Foxp3 (C) and STAT 3 (D) in isolated CD4^+^ cells was measured in all the groups. CD4^+^ cells of five animals were pooled in each group for RNA isolation and to run real-time PCR assay. Data represent three independent experiments and expressed as mean ± SEM with 95% confidence interval. Statistical analysis was performed by ANOVA method followed by the Newman–Keuls test of significance using Prism version 3.0 software.

The differentiation of Th17 cells is regulated by various factors like STAT 3 (signal transducer and activation of transcription), ROR-α, ROR-γt and Foxp3. Transcript levels of these factors were determined in purified CD4^+^ cells from the bone marrow. In comparison to the controls, mRNA levels of ROR-α (P<0.001), ROR-γt (P<0.001) and STAT3 (P<0.001) were higher in the Ovx group (Fig. 5 A, B, and D). Treatment of E2 to Ovx mice had no difference in ROR-α and STAT3 mRNA levels when compared with sham group (Fig. 4A, E). mRNA levels of ROR-γt was although decreased (P<0.001) in the E2 treated Ovx mice compared to the Ovx group, but was higher than the sham group (P<0.05). Expression of Foxp3 (P<0.001) gene was significantly decreased in Ovx mice compared to sham group while E2 treatment to Ovx mice significantly upregulated Foxp3 (P<0.01) expression (Fig. 5C).

### Effect of IL-17 Functional Block on Serum CTx Level and OPG/RANKL Transcripts

**Figure 6:**
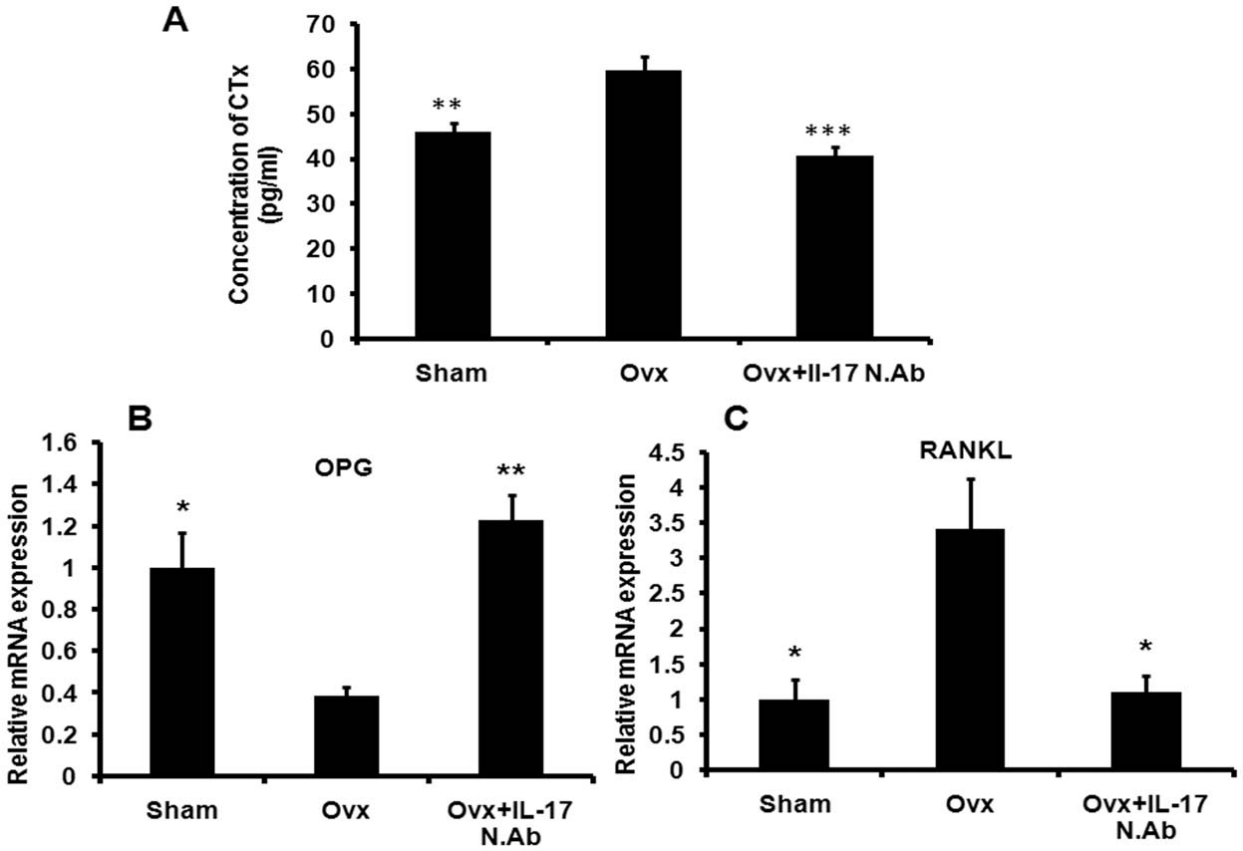
IL-17 neutralization reduces serum CTx level in Ovx mice and decreases RANKL expression in femur trabecular bone. (A) After treatment animals were sacrificed and serum samples were collected from all the groups. C-terminal telopeptides of type I collagen (CTx) level was measured in all the groups by ELISA. ELISA was conducted in six replicates in each group. (B) mRNA level of OPG in the trabecular region of femur bone was studied in all the groups (method was explained in the material and method section). (C) mRNA level of RANKL in the trabecular region of femur bone was studied in all the groups. RNA from four femur bones was pooled for the study of OPG and RANKL in each group. Data represent three independent experiments and expressed as mean ± SEM with 95% confidence interval. Statistical analysis was performed by ANOVA method followed by the Newman–Keuls test of significance using Prism version 3.0 software.

In comparison to the sham group, serum CTx level was increased in the Ovx group (P<0.01), and the levels were not different between the sham and Ovx mice treated with neutralizing IL-17 antibody (NIL-17 mAb) (Fig. 6A). Bones devoid of BMC were harvested from the various mice groups. In comparison to the sham mice, the mRNA levels of OPG were decreased (P<0.05) (Fig. 6B) and RANKL were increased (P<0.05) (Fig. 6C) in the long bones of the Ovx mice. There were no differences in the OPG and RANKL transcript levels between the sham and Ovx + NIL-17 mAb groups (Fig. 6B–C).

### Effect of IL-17 Functional Block on Trabecular Microarchitecture of Ovx Mice

**Figure 7:**
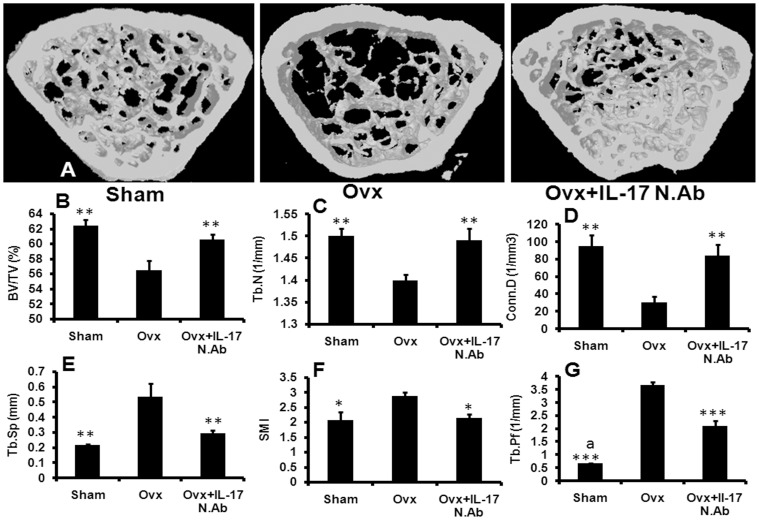
IL-17 neutralization blocks Ovx induced bone loss and improves trabecular microarchitecture in femur. (A) After treatment animals were sacrificed and long bones were collected in 70% isopropanol. (A) BV/TV, (B) Tb.N., (C) Conn.D, (D) Tb.Sp, (E) SMI and (F) Tb.Pf. Blocking of IL-17 with NIL-17mAb restores Ovx induced alterations in femur trabecular region. Six mice were taken in each group for the study. Data expressed as mean ± SEM with 95% confidence interval. Statistical analysis was performed by One way-ANOVA nonparametric method followed by the Newman–Keuls test of significance using Prism version 3.0 software. ^***^ *P*<0.001, ^**^ *P*<0.01 and ^*^ *P*<0.05 compared with Ovx animals; ^a^ *P*<0.001 compared between sham and Ovx + IL-17 N.Ab.

In gross observation by 3D-µCT, deterioration of the trabecular architecture due to destruction of trabecular bone of femur and tibia were readily observed in Ovx group compared with sham group (Fig. 7A). Femoral response to various treatments was quantified. The Ovx group had reduced BV/TV (P<0.01) (Fig. 7B), Tb.N (P<0.01) (Fig. 7C) and Conn.Dn (P<0.01) (Fig. 7D); and increased Tb.Sp (P<0.01) (Fig. 7E), Tb.Pf (P<0.001) (Fig. 7F) and SMI (P<0.05) (Fig. 7G). There were no differences in the trabecular parameters except Tb.pf between the sham and Ovx mice treated with NIL-17 mAb. Tb.pf was lower in the sham group compared with the Ovx + NIL-17 mAb group (p<0.001).

**Figure 8:**
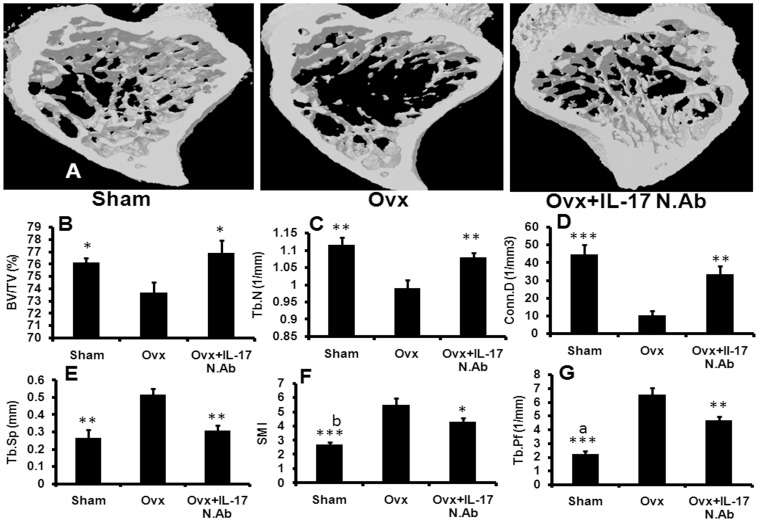
IL-17 neutralization blocks Ovx induced bone loss and improves trabecular microarchitecture in tibia. (A) After treatment animals were sacrificed and long bones were collected in 70% isopropanol. (A) BV/TV, (B) Tb.N., (C) Conn.D, (D) Tb.Sp, (E) SMI and (F) Tb.Pf. Blocking of IL-17 with NIL-17mAb restores Ovx induced alterations in tibial trabecular region. Six mice were taken in each group for the study. Data expressed as mean ± SEM with 95% confidence interval. Statistical analysis was performed by One way-ANOVA nonparametric method followed by the Newman–Keuls test of significance using Prism version 3.0 software. ^***^ *P*<0.001, ^**^ *P*<0.01 and ^*^ *P*<0.05 compared with Ovx animals; ^a^ *P*<0.001 and ^b^ *P*<0.01 compared between sham and Ovx + IL-17 N.Ab.

Paralleling the femur trabecular data, tibia metaphysis in Ovx mice presented with huge microarchitectural loss compared to the sham group (Fig. 8A). Ovx mice had decreased BV/TV (P<0.05) (Fig. 8B), Tb.N (P<0.01) (Fig. 8C), and Conn.Dn (P<0.001) (Fig. 8D) and increased Tb.Sp (P<0.01) (Fig. 8E), Tb.Pf (P<0.001) (Fig. 8F) and SMI (P<0.001) (Fig. 8G). There was no difference in BV/TV, Tb.sp, Tb.N, and Conn.D between the sham and Ovx + NIL-17 mAb groups. Tb.Pf (p<0.001) and SMI (P<0.01) were lower in the sham group compared with the Ovx + NIL-17 mAb group. However, compared with the Ovx group, Tb.Pf (p<0.01) and SMI (P<0.05) were lower in the Ovx + NIL-17 mAb.

### Effect of IL-17 Functional Block on the Mineralization of Bone Marrow Stromal Cells *ex vivo*

**Figure 9:**
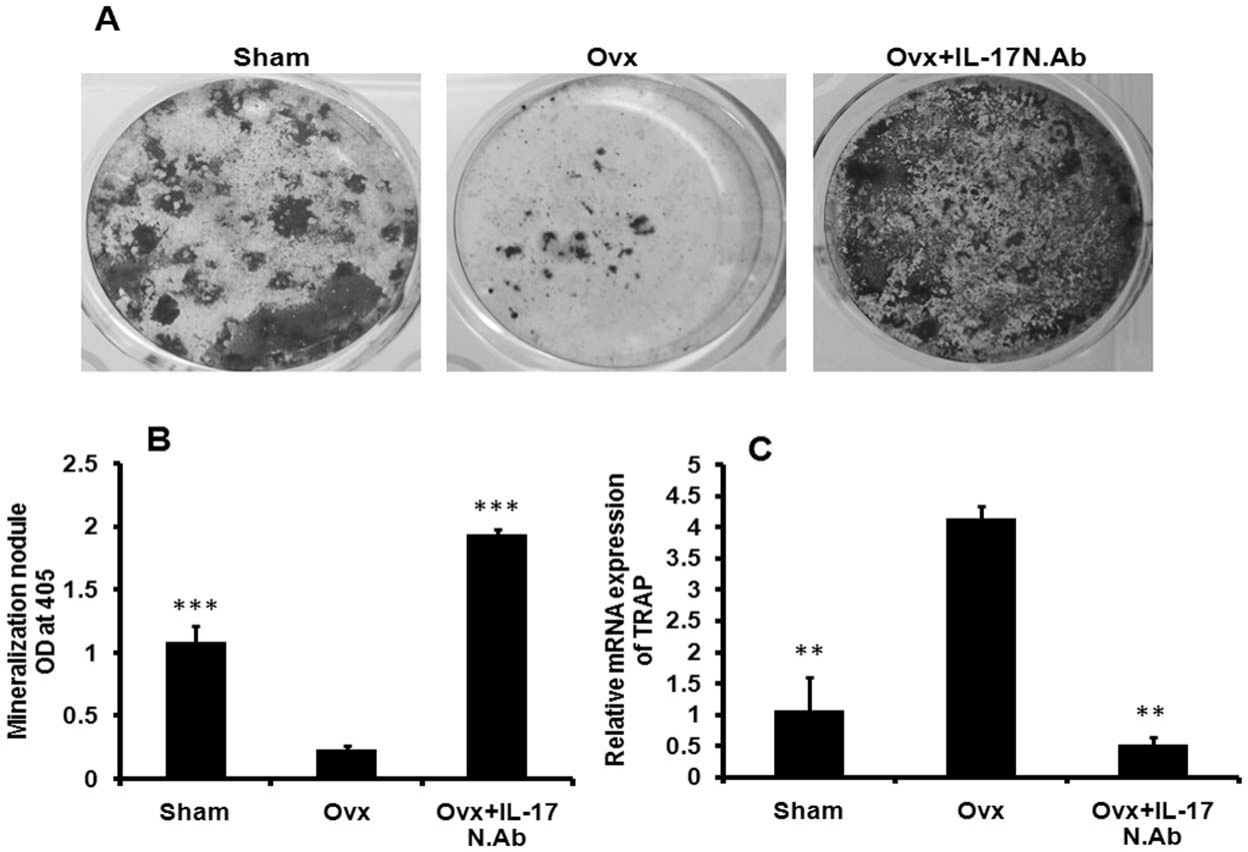
NIL-17 mAb treatment to Ovx mice promotes mineral nodule formation and inhibits osteoclastogenesis in bone marrow microenvironment. (A) Representative images show mineralization nodules formed in the BMCs harvested from the long bones of all groups of mice. (B) Quantitative measurement of mineralization nodules in all the groups were performed by using alizarin red staining. (C) *Ex-vivo* osteoclastogenesis was performed in BMCs of all the groups. After five days total RNA was isolated and real-time PCR was performed to assess the expression of TRAP gene. Data represent three independent experiments and expressed as mean ± SEM with 95% confidence interval. Statistical analysis was performed by ANOVA method followed by the Newman–Keuls test of significance using Prism version 3.0 software.

BMC harvested from the various groups were cultured in osteogenic medium to induce the formation of mineralized nodules (Fig. 9A). Mineralized nodules formed in the BMC derived from Ovx mice were found to be significantly fewer than the sham group (P<0.001) and Ovx + NIL-17 mAb groups (P<0.001) (Fig. 9B). For ex vivo osteoclastogenesis, BMC were harvested from the various mice groups. In comparison to the sham mice, BMCs from Ovx mice responded to osteoclast differentiation stimuli (RANKL + M-CSF) with higher levels of TRAP positive cells (P<0.01) (Fig. 9C). NIL-17 mAb treatment to Ovx mice reduces TRAP positive cells (Fig. 9C) (P<0.01).

## Discussion

Our study demonstrated that IL-17 directly stimulated osteoclast differentiation and this effect was reversed in BMCs pre-treated with E2. In osteoblasts, IL-17 inhibited the bone matrix minerlization and production of osteoclastogenic cytokines, and again, E2 antagonized this effect. In Ovx mice, there is increased proliferation of Th17 cells, which in turn is induced by differentiation of naïve CD4^+^ T cells into Th17 cells by increased expression of ROR-γt and ROR-α, the receptors responsible for Th-17 differentiation of CD4^+^ cells. Amplified differentiation of Th17 cells leads to increased levels of circulating IL-17. E2 supplementation to Ovx mice completely reversed each of these changes. Finally, systemic neutralization of IL-17 in Ovx mice prevented the bone loss phenotypes, including microarchitectural deterioration, increased bone resorption marker and changes in the cellular and molecular parameters of bone cells, suggesting a vital role of IL-17 in mediating E2 deficiency-induced bone loss.

E2 most potently inhibits osteoclast production and function, and this is the primary mechanism by which it conserves skeleton during the reproductive life of mammals in females [52]. Exogenous addition of IL-17 in the BMC cultures promoted osteoclast differentiation induced by RANKL + M-CSF, suggesting a direct stimulatory effect of IL-17 in osteoclast production. Pre-treatment of E2 abrogated IL-17-induced osteoclast differentiation, suggesting that E2 negatively regulated IL-17 effect. These observations were contrary to the results obtained by Kitami *et al* [53] who reported that IL-17A suppresses the expression of bone resorption-related proteinases and osteoclast differentiation via IL-17RA or IL-17RC receptors in RAW264.7 cells. However, in this study we have used bone marrow cells derived from long bones instead of a cell line and cell lines may differ from *in vivo* situations. Moreover, our observations are corroborated by several studies which show that IL-17 is a potent stimulator of osteoclastogenesis [20], [30], [54].

**Table 1:** Sequences of real-time PCR primers.

GeneName	Primer Sequence	Accession Number
**GAPDH**	P1-5′- AGCTTGTCATCAACGGGAAG -3′P2-5′- TTTGATGTTAGTGGGGTCTCG -3′	NM_008084.2
**TNF-α**	P1-5′- TGCCTATGTCTCAGCCTCTTC-3′P2-5′- GAGGCCATTTGGGAACTTCT-3′	NM_013693.2
**IL-6**	P1-5′- GCTACCAAACTGGATATAATCAGGA-3′P2-5′- CCAGGTAGCTATGGTACTCCAGAA-3′	NM_031168.1
**OPG**	P1-5′-GTTTCCCGAAGGACCACAAT-3′P2-5′-CCATTCAATGATGTCCAGGAG-3′	U94331.1
**RANKL**	P1-5′- TGAAGACACACTACCTGACTCCTG-3′P1-5′- CCACAATGTGTTGCAGTTCC-3′	AF019048.1
**TRAP**	P1-5′-GGTCAGCAGCTCCCTAGAAG-3′P2-5′-GGAGTGGGAGCCATATGATTT-3′	NM_001102405.1
**RANK**	P1-5′-AGAGGCATTATGAGCATCTCG-3′P2-5′-GGAGTGCACTTAGAGGACAGGT-3′	AF019046.1
**IL-17A**	P1-5′-CAGGGAGAGCTTCATCTGTGT-3′P2-5′-GCTGAGCTTTGAGGGATGAT-3′	NM_010552.3
**ROR-α**	P1-5′-TTACGTGTGAAGGCTGCAAG-3′P2-5′-GGAGTAGGTGGCATTGCTCT-3′	NM_013646.1
**ROR-γt**	P1-5′-CACTGCCAGCTGTGTGCT-3′P2-5′-TGCAAGGGATCACTTCAATTT-3′	AF163668.1
**IL-17R**	P1-5′- TGGGATCTGTCATCGTGCT-3′P2-5′- ATCACCATGTTTCTCTTGATCG-3′	NM_008359.2
**STAT 3**	P1-5′- GGAAATAACGGTGAAGGTGCT-3′P2-5′- CATGTCAAACGTGAGCGACT-3′	NM_213659.2
**FOXP3**	P1-5′- AGAAGCTGGGAGCTATGCAG-3′P1-5′- GCTACGATGCAGCAAGAGC-3′	BC132333.1

Studies by Kocic et al [55] and Huang et al [54] have shown that IL-17 has a positive effect on osteoblast differentiation. However, in our study we observed that IL-17 treatment to MCO leads to decreased mineralization. Our observations are supported by that of Won et al [24] who conducted a study in TallyHo/JngJ (TH) mice, a polygenic model of type II diabetes which spontaneously develops bone deformities with osteoporotic features. In these mice CD4^+^ T cells are biased towards Th17 cell differentiation. It was seen that osteoblast-specific bone forming markers like osteocalcin and osteoprotegerin were decreased, whereas osteoclast-driven bone resorption markers such as IL-6 and RANKL were significantly elevated in the bone marrow and blood of these mice. In our study, we also observed IL-17 mediated inhibition of osteoblast differentiation and increase in RANKL expression in calvarial osteoblast cells. Again, our studies make use of primary cell culture system unlike the cell line system used in studies by Kocic and Huang et al [54], [55]. Pre-treatment with E2 inhibited IL-17 mediated decrease in mineralization.

It is well known that IL-17 induces the expression of several cytokines like IL-1, IL-6 and TNF-α known to contain nuclear factor kappa B (NF-*k*B) binding sites in their promoter [51]. We observed that IL-17 treatment led to increased production of osteoclastogenic cytokine like TNF-α and IL-6 from MCO. Studies by Yago et al [30] have shown that blockade of infliximab, the TNF-α monoclonal antibody abrogated the effect of IL-17 on osteoclastogenesis. Thus, it is possible that IL-17 induces TNF-α production in osteoblasts which may in turn be responsible for IL-17 mediated effects. These IL-17 mediated effects were blocked by E2 which is known to repress the transcription of NF-kB target genes expressed by osteoblasts. E2 also abrogated the IL-17 induced expression of IL17 receptor and NF-kB translocation in osteoblasts thus inhibiting the downstream signalling mediated by binding of IL-17 to its receptor. Together, these data suggested that IL-17 could adversely impact bone by simultaneous effect of augmenting osteoclast production and inhibiting osteoblast function and E2 could reverse these effects.

*In vivo*, IL-17 is regulated by E2 as evidenced by increased Th17 CD4^+^ population in the BM and circulating IL-17 in Ovx mice. Reversal of these effects by E2 suggested that the hormone negatively regulated Th17 CD4^+^ population in the BM to reduce systemic IL-17 levels. Additionally, IL-17 mRNA levels in the CD4^+^ cells were inversely regulated by E2 status. This led us to investigate the role of E2 in Th17 cell lineage differentiation. The differentiation of Th17 cells is initiated by signal transducer and activator of transcription 3 (STAT3) which induces the expression of retinoic-acid-receptor-related orphan receptor-α (ROR-α) and ROR-γt [56]. These two factors establish the Th17-cell-associated gene-expression programme, leading to the production of IL-17. In addition, the transcription factor forkhead box P3 (Foxp3), which is induced by transforming growth factor-β (TGF-β) signaling, antagonizes the Th17-cell developmental programme [56]. We observed that while Ovx leads to an upregulation of STAT3, ROR-α and ROR-γt and downregulated Foxp3 transcript levels in purified CD4^+^ cells from BMCs, E2 reversed these effects. Thus, E2 negatively regulates Th17 differentiation, thereby reducing IL-17 cytokine production and subsequent bone loss. Guery etal [57] have also reported that estrogen receptor α signalling in T cells is required for E2 mediated Th17 cell differentiation and protection against Experimental Autoimmune Encephalomylitis. Besides, our data is strengthened by studies of Luo et al [58] where estrogen enhances the functions of CD4^+^CD25^+^Foxp3^+^ regulatory T cells that suppress osteoclast differentiation and bone resorption *in vitro* and Foxp3 is known to antagonize the Th17 development programme.

As estrogen deficiency induced the differentiation of IL-17 secreting Th17 cells, the next step was to study whether IL-17 functional block has any effect on Ovx induced bone loss. Thus, we made use of neutralizing antibody against IL-17 (NIL-17 mAb) in Ovx mice and studied the skeletal impact. One of the most important markers for bone resorption is the serum CTx levels which are peptide fragments from collagen degradation by cathepsin enzyme that is released by osteoclasts through bone resorption. These fragments enter the circulation and serve as sensitive and specific measures of bone resorption [59], [60]. As expected, the CTx levels were raised in the Ovx mice but reduced in the NIL-17 mAb group, thus confirming that the bone resorbing effect of IL-17 in Ovx mice is due to increased osteoclast function. Enhanced TRAP positive cells in the BMC of Ovx mice were suppressed by NIL-17 mAb, which further attested to the osteoclast promoting action of IL-17 *in vivo*.

Reduction in OPG and increase in RANKL in Ovx bones favours osteoclast differentiation and activation, and promotes bone loss [61]. E2 enhances osteoblastic OPG and decreases RANKL expression. Accordingly, in Ovx bones there was reduced OPG and increased RANKL expression. However NIL-17 mAb treatment to Ovx mice maintained the expression of both the genes to the sham level. These results indicate that IL-17 has a strong catabolic effect by increasing osteoclast production directly as well as indirectly through an alteration in OPG/RANKL system from the osteoblasts. In addition, osteoblast differentiation from the BM stromal cells was reduced by IL-17 *in vitro* and *in vivo*, suggesting its anti-anabolic effect. This effect was however inhibited by NIL-17 mAb treatment to Ovx mice *in vivo*.

Activation of the catabolic pathway together with the anti-osteoblastic effect triggers erosion of trabecular bone and deterioration of its microarchitecture in Ovx bones [62]. At the distal femoral epiphysis and tibia metaphysis, NIL-17 mAb remarkably mitigated Ovx-induced microarchitectural loss. The stability of trabecular bone is importantly dependent on structural parameters determined by SMI and Tb.pf. In contrast to Ovx + vehicle group, preferred plate-like structure (lower SMI) and more concave trabecular surface (lower Tb.pf), presented a more compact and well connected spongy lattice in NIL-17 mAb treated mice. The deterioration of bone mass and structure is generally greater in the tibia metaSphysis over femur epiphysis, and NIL-17 mAb was effective in attenuating the adverse trabecular changes in the tibia, confirming the *in vivo* relevance of neutralizing IL-17 in Ovx-induced loss of cancellous bone. These results were contrary to that reported by Goswami et al [26] where Ovx IL-17 receptor knockout mice were more susceptible to Ovx-induced bone loss than controls suggesting a bone protective role for IL-17 receptor signalling. However, our results are in agreement with DeSelm et al who observed that Ovx induced bone loss is prevented by an antibody targeting the IL-17 cytokine [28].

In conclusion, the present study provides evidence that IL-17 - a member of Th17 cytokine - promotes bone loss by favoring osteoclast production and inhibiting osteoblast differentiation, and whose production is under the negative regulation of E2. Moreover, an inhibition of IL-17 having bone sparing effect under Ovx by antibody approach could form the basis for using humanized antibody against this cytokine towards the treatment of postmenopausal osteoporosis.

## Materials and Methods

### Reagents and Chemicals

Cell culture media and supplements were purchased from Invitrogen (Carlsbad, CA). All fine chemicals including 17-β Estradiol and primers were purchased from Sigma-Aldrich (St. Louis, MO). IL-17 neutralizing antibody and all other antibodies used for FACS studies were purchased from BD Biosciences (Mississauga, ON, CA). IL-17 and CTx ELISA kit was purchased from Immunodiagnostic systems Ltd. UK.

### *In vitro* and *Ex vivo* Osteoclastogenesis

*In vitro* osteoclastogenesis was studied using bone marrow cells of healthy mice and ex-vivo osteoclastogenesis was evaluated using bone marrow cells from all the experimental groups using our previously published protocol [29]. Briefly, mouse bone marrow cells were flushed from long bones using α-MEM supplemented with 10% FCS. After overnight culture in this medium, non-adherent bone marrow cells were seeded in 6-well plates at a density of 500,000 cells/well and cultured for 5–6 days in α-MEM containing 10% FCS, 50 ng/ml RANKL, and 10 ng/ml MCSF and IL-17 at concentration of 10 and 100 ng/ml [30]. Medium was replaced on the third day. After 6 days of culture, cells were washed in PBS and then fixed in 4% para-formaldehyde for 10 min. This was followed by staining of tartrate resistant acid phosphatase cells using the standardized protocol [31] or analysis of TRAP and RANK mRNA levels by real-time quantitative PCR.

### Effect of E2 on IL-17 Mediated Mineralization and Osteoclastogenic Cytokine Production in Mouse Calvarial Osteoblast Cells

Mouse calvarial osteoblasts (MCO) were obtained following our previously published protocol of sequential digestion [29], [32], [33], [34]. Briefly, calvaria from ten to twelve 1- to 2-days old Balb/c mice (both sexes) were pooled. After surgical isolation from the skull and the removal of sutures and adherent mesenchymal tissues, calvaria were subjected to five sequential (10–15 min) digestions at 37^°^C in a solution containing 0.1% dispase and 0.1% collagenase P. Cells released from the second to fifth digestions were collected, centrifuged, resuspended, and seeded in T-25 cm^2^ flasks in α-MEM containing 10% FCS and 1% penicillin/streptomycin (complete growth medium). To study bone mineralization, cells were seeded at confluence at a density of 5×10^3^ cells per well in 12 well plates and were cultured up to 21 days. Treatment with E2 at 10^−9^M [9], [35] concentration and IL-17 cytokine at 100 ng/ml [30] was given to the cells. At the end of the experiment, cells were washed with PBS and fixed with 4% para-formaldehyde in PBS for 15 min. The fixed cells were stained with 40 mM (pH 4.5) Alizarin Red-S for 30 min followed by washing with water [29]. For quantification of staining, 800 µl of 10% (v/v) acetic acid was added to each well, and plates were incubated at room temperature for 30 min with shaking. The monolayer, now loosely attached to the plate, was then scraped from the plate with a cell scraper and transferred with 10% (v/v) acetic acid to a 1.5-ml tube. After vortexing for 30s, the slurry was overlaid with 500 µl mineral oil (Sigma–Aldrich), heated to exactly 85°C for 10 min, and transferred to ice for 5 min. The slurry was then centrifuged at 20,000×*g* for 15 min and 500 µl of the supernatant was removed to a new tube. Then 200 µl of 10% (v/v) ammonium hydroxide was added to neutralize the acid. In some cases, the pH was measured at this point to ensure that it was between 4.1 and 4.5. OD (405 nm) of 150 µl aliquots of the supernatant were measured in 96-well format using opaque-walled, transparent-bottomed plates [36], [37].

For studying the expression profile of inflammatory cytokines, cells were seeded in six well plates in 10% α-MEM medium. On confluency these cells were treated with or without test agents for 24 h. After 24 h cells were collected in TRIZOL agent for RNA isolation and qPCR.

### Effect of E2 on IL-17 Mediated NF-κB Translocation

For analysis of NF-κB nuclear translocation at protein level, nuclear extract was prepared as previously described [38]. Briefly, after treatment of the osteoblast cells as indicated above, cells were washed in 1 ml of ice-cold PBS, centrifuged at 400×g for 5 min, resuspended in 400 µl of ice-cold hypotonic buffer (10 mM HEPES/KOH, 2 mM MgCl2, 0.1 mM EDTA, 10 mM KCl, 1 mM DTT, 0.5 mM PMSF, pH 7.9), left on ice for 10 min, vortexed, and centrifuged at 15,000×g for 30 s. Pelleted nuclei were resuspended in 50 µl of ice-cold saline buffer (50 mM HEPES/KOH, 50 mM KCl, 300 mM NaCl, 0.1 mM EDTA, 10% glycerol, 1 mM DTT, 0.5 mMPMSF, pH 7.9), left on ice for 20 min, vortexed, and centrifuged at 15,000×g for 5 min at 4°C. Supernatant was collected and the supernatant that contained nuclear proteins was frozen in liquid nitrogen and stored at −80°C until use. For western blot analysis, samples were electrophoresced using 10% SDS-PAGE gel electrophoresis, as described before [32], and then transferred to a nitrocellulose membrane. NF-κB nuclear translocation was determined using anti-p65 antibody (Santa Cruz Biotech, USA). Immunodetection was done using an enhanced chemiluminescence detection kit (Amersham Pharmacia, USA).

### *In vivo* Studies

The study was conducted in accordance with current legislation on animal experiments and was approved by the Institutional animal ethics committee, Central Drug Research Institute [CPSCEA Registration Number 34/1999 dated 11.3.99 (Extended upto 2012). Approval reference number IAEC/2010/127/Renew 01 dated 9.2.2012]. Adult Balb/c mice (9–10 week-old) were used for the studies [39], [40], [41], [42]. All mice were housed at 25°C, in 12-hour light: 12-hour dark cycles. Normal chow diet and water were provided ad libitum. For the first set of experiments ten mice per group were taken. The groups were: sham-operated (ovary intact) mice, Ovariectomized (Ovx) and Ovx+0.01 mg/kg E2 [9], [35]. The treatment was given by oral gavage for six weeks. In the second set of experiment six mice per group were used. The groups were: sham-operated, Ovx and Ovx +100 ng/mice twice/week subcutaneously IL-17 neutralizing antibody group [43], [44], [45]. Sham operated group served as the positive control group and was given vehicle (IgG in normal saline). All treatments were given by intra-peritoneal route and continued for 4 weeks. At the completion of study, animals were autopsied. After autopsy, bones were dissected and the bone marrow (BM) was flushed out. Total lymphocytes from the BM were isolated by using Hisep LSM 1084 (Himedia) by means of density (1.08460.0010 g/ml) gradient centrifugation technique [9], [35], [46], [47]. Long bones were kept in 70% isopropanol for µCT study. Pure CD4^+^, cells were retrieved from the BM by positive selection using microbeads based isolation by MACS separator according to the manufacturer’s protocol (EasySep Biotin Selection Kit, Stem Cell Technologies Inc., Vancouver, BC, CA). These purified cells were then collected in Trizol for Real time PCR (qPCR). Serum was collected for ELISA. Serum TNF-α and C-terminal telopeptides of type I collagen (CTx) were measured in all the groups by using ELISA kit according to manufacturer’s instructions.

### Intracellular Staining of IL-17

Intracellular staining was done as per manufacturer’s instructions. In brief, single cell suspension of the BM was prepared in PBS. Then cells were centrifuged at 500 rpm for 5 minutes. Supernatant was discarded and cells were suspended in 1 ml of PBS. Cells were then stimulated for 6 h with 10 ng/ml PMA, 250 ng/ml ionomycin, and 2 µM monensin for 6 h. After harvesting, cells were first subjected to surface marker staining followed by intracellular Il-17 staining on ice with anti mouse CD3, CD4 and IL-17 antibodies (PE conjugated anti-mouse IL-17, APC conjugated anti-mouse CD4 and FITC conjugated anti-mouse CD3) to assess the percentage of CD4^+^IL-17^+^ cells in CD3^+^ cells. Specificity of immunostaining was ascertained by the background fluorescence of cells incubated with Ig isotype controls. After surface and intracellular staining, cells were washed twice with PBS and transferred to FACS tubes for analysis. FACS Caliber and FACS Arya (BD Biosciences Mississauga, ON, CA) were used to quantify the percentage of IL-17^+^ CD4 T cells in CD3^+^ cells in all the groups [48].

### Total RNA Isolation and Quantitative Real-Time-PCR

Total RNA was extracted from isolated CD4^+^ T cells of all the *in vivo* groups and *in vitro* cultured cells using Trizol (Invitrogen). cDNA was synthesized from 1 µg total RNA with the Revert AidTM H Minus first strand cDNA synthesis kit (Fermentas, USA). SYBR green chemistry was used for quantitative determination of the mRNAs for IL-17A, ROR-α, RORγt, STAT3, Foxp3, IL-17R, RANK, RANKL, TRAP, OPG and a housekeeping gene, GAPDH, following an optimized protocol. The design of sense and antisense oligonucleotide primers was based on published cDNA sequences using the Universal probe library (Roche Diagnostics, USA). Primer sequences are given in table 1. For real-time PCR, the cDNA was amplified with Light Cycler 480 (Roche Diagnostics Pvt. Ltd.).

The double-stranded DNA-specific dye SYBR Green I was incorporated into the PCR buffer provided in the Light Cycler 480 SYBER green I master (Roche Diagnostics Pvt. Ltd.) to allow for quantitative detection of the PCR product in a 20 µl reaction volume. The temperature profile of the reaction was 95°C for 5 min, 40 cycles of denaturation at 94°C for 2 min, and annealing and extension at 62°C for 30 sec, extension at 72°C for 30 sec. GAPDH was used to normalize differences in RNA isolation, RNA degradation, and the efficiencies of the reverse transcription.

### Microcomputed Tomography (µCT) in Long Bones

Microcomputed tomographic (µCT) determination of excised bones was carried out using the Sky Scan 1076 CT scanner (Aartselaar, Belgium) using previously published protocol [29], [35]. Femora and tibiae were dissected from the animals after they were killed, cleaned of soft tissue and fixed before storage in alcohol. The samples were scanned in batches of three at a nominal resolution (pixels) of 18 µm. Reconstruction was carried out using a modified Feldkamp algorithm using the Sky Scan Nrecon software, which facilitates network distributed reconstruction carried out on personal computer running simultaneously. The x-ray source was set at 70 kV and 100 mA, with a pixel size of 18 µm. A hundred projections were acquired over an angular range of 180°. The image slices were reconstructed using the cone-beam reconstruction software version 2.6 based on the Feldkamp algorithm Skyscan). Parameters like fractional bone volume (bone volume per tissue volume, BV/TV), trabecular number (Tb.N), separation (Tb.Sp), and structural indices including structure model index (SMI), connection sensitivity (Conn.D), and trabecular pattern factor (Tb.Pf) were calculated. Three-dimensional parameters were based on analysis of a Marching cubes type model with a rendered surface.

### Measurement of Bone Relevant Serum CTx

Following our previously published protocol [49], serum CTx level was determined by enzyme-linked immunosorbent assay kits purchased from Immunodiagnostic Systems Inc.

### *Ex vivo* Mineral Nodule Formation Assay

For *ex vivo* experiment, bone marrow cells from all the experimental groups were flushed and cultured in osteoblast differentiation medium (10 nM dexamethasone, 10 mM β-glycerophosphate and 50 µg/ml ascorbic acid) for 18days. Alizarin red-S stain was used for staining mineralized nodules followed by extraction of the stain for quantitation [29].

### Statistical Analysis

Data are expressed as mean ± S.E.M. The data obtained in experiments were subjected to One-way ANOVA followed by Newman–Keuls test of significance using Prism version 3.0 software. Qualitative observations have been represented following assessments made by three individuals blinded to the experimental designs.

## Supporting Information

Table S1 **Micro computed tomographic (µ-CT) determination of excised femora were carried out using the sky Scan 1076 KCT scanner (Artselaar, Belgium) Percent bone volume (BV/TV %), Trabecular separation Tb.Sp (mm), Trabecular number Tb.N (1/mm), Trabeculat pattern factor Tb.Pf were calculated by the mean intercept length method.** N = 10 mice/group; data are presented as mean± SEM *P<0.0, **P<0.01, ***P<0.001.
(DOC)
