# Supplementary material for: Estrogen Deficiency Induces the Differentiation of IL-17 Secreting Th17 Cells: A New Candidate in the Pathogenesis of Osteoporosis
Source: PLoS One. 2012 Sep 10;7(9):e44552. doi: 10.1371/journal.pone.0044552 (PMC3438183; doi:10.1371/journal.pone.0044552)
Supplement: Table S1 — Micro computed tomographic (µ-CT) determination of excised femora were carried out using the sky Scan 1076 KCT scanner (Artselaar, Belgium) Percent bone volume (BV/TV %), Trabecular separation Tb.Sp (mm), Trabecular number Tb.N (1/mm), Trabeculat pattern factor Tb.Pf were calculated by the mean intercept length method. N = 10 mice/group; data are presented as mean± SEM *P<0.0, **P<0.01, ***P<0.001. (DOC) [file pone.0044552.s001.doc]

**Table-S1**

µ-CT analysis of femur trabiculae of Balb/C Mice

| **Parameters** | **Sham** | **Ovx** | **Ovx+17β-E2**  **0.01mg/kg/d** |
| --- | --- | --- | --- |
| **Percent bone volume**  **(BV/TV %)** | 22.7±7.66*** | 7.34±0.302 | 18.84±6.88*** |
| **Trabecular separation**  **Tb.Sp (mm)** | 0.23±0.03*** | 0.52±0.12 | 0.38±0.11*** |
| **Trabecular number**  **Tb.N (1/mm)** | 2.83±0.75*** | 1.11±0.07 | 2.19±0.67*** |
| **Trabeculat pattern factor**  **Tb.Pf** | 10.24±5.72*** | 18.70±2.9 | 11.20±4.40*** |
